# Comprehensive Evaluation of the MBT STAR-BL Module for Simultaneous Bacterial Identification and β-Lactamase-Mediated Resistance Detection in Gram-Negative Rods from Cultured Isolates and Positive Blood Cultures

**DOI:** 10.3389/fmicb.2018.00334

**Published:** 2018-02-23

**Authors:** Annie W. T. Lee, Johnson K. S. Lam, Ricky K. W. Lam, Wan H. Ng, Ella N. L. Lee, Vicky T. Y. Lee, Po P. Sze, Rahim Rajwani, Kitty S. C. Fung, Wing K. To, Rodney A. Lee, Dominic N. C. Tsang, Gilman K. H. Siu

**Affiliations:** ^1^Department of Health Technology and Informatics, The Hong Kong Polytechnic University, Hung Hom, Hong Kong; ^2^Department of Pathology, Queen Elizabeth Hospital, Kowloon, Hong Kong; ^3^Department of Pathology, Pamela Youde Nethersole Eastern Hospital, Hong Kong, Hong Kong; ^4^Department of Pathology, United Christian Hospital, Kowloon, Hong Kong; ^5^Department of Pathology, Princess Margaret Hospital, Kowloon, Hong Kong

**Keywords:** MBT STAR-BL, MALDI-TOF MS, drug resistance, bacterial, drug hydrolysis test, beta-lactamases, blood culture

## Abstract

**Objective:** This study evaluated the capability of a MALDI Biotyper system equipped with the newly introduced MBT STAR-BL module to simultaneously perform species identification and β-lactamase-mediated resistance detection in bacteremia -causing bacteria isolated from cultured isolates and patient-derived blood cultures (BCs).

**Methods:** Two hundred retrospective cultured isolates and 153 prospective BCs containing Gram-negative rods (GNR) were collected and subjected to direct bacterial identification, followed by the measurement of β-lactamase activities against ampicillin, piperacillin, cefotaxime, ceftazidime, and meropenem using the MBT STAR-BL module. The results and turnaround times were compared with those of routine microbiological processing. All strains were also characterized by beta-lactamase PCR and sequencing.

**Results:** Using the saponin-based extraction method, MALDI-TOF MS correctly identified bacteria in 116/134 (86.6%) monomicrobial BCs. The detection sensitivities for β-lactamase activities against ampicillin, piperacillin, third-generation cephalosporin and meropenem were 91.3, 100, 97.9, and 100% for cultured isolates, and 80.4, 100, 68.8, and 40% for monomicrobial BCs (*n* = 134) respectively. The overall specificities ranged from 91.5 to 100%. Furthermore, the MBT STAR-BL and conventional drug susceptibility test results were concordant in 14/19 (73.7%) polymicrobial cultures. Reducing the logRQ cut-off value from 0.4 to 0.2 increased the direct detection sensitivities for β-lactamase activities against ampicillin, cefotaxime and meropenem in BCs to 85.7, 87.5, and 100% respectively. The MBT STAR-BL test enabled the reporting of β-lactamase-producing GNR at 14.16 and 47.64 h before the interim and final reports of routine BCs processing, respectively, were available.

**Conclusion:** The MALDI Biotyper system equipped with the MBT STAR-BL module enables the simultaneous rapid identification of bacterial species and β-lactamase-mediated resistance from BCs and cultured isolates. Adjustment of the logRQ cut-off value to 0.2 significantly increased the detection sensitivities for clinically important drug-resistant pathogens.

## Introduction

Sepsis is a major cause of infectious disease-associated morbidity and mortality (Fleischmann et al., 2016). Proper initial antibiotic therapy is a crucial parameter for improvement of patient outcomes (Kumar et al., 2006; Dellinger et al., 2008). Empirical treatment must be administrated at the time of sepsis diagnosis, and the regimen should be adjusted if necessary when bacterial identification and drug susceptibility results are available (Kang et al., 2005; Dellinger et al., 2008; Kumar, 2011). Matrix-assisted laser desorption ionization-time of flight mass spectrometry (MALDI-TOF MS) is particularly useful for direct identification of causative agents of bacteremia, which can be inducers of sepsis, within the same day of a positive blood culture (BC) broth result (Schubert et al., 2011; Wuppenhorst et al., 2012; Chen et al., 2013; Clerc et al., 2013). However, predictions of drug susceptibility patterns based on bacterial identification alone have become inaccurate, given the increasing incidence of multidrug-resistance among Gram-negative bacteria (Paterson, 2006; Livermore, 2012).

Recent studies demonstrated the feasibility of using MALDI-TOF MS to predict β-lactam resistance through detection of hydrolytic β-lactam substrates produced by bacterial β-lactamases (Burckhardt and Zimmermann, 2011; Hrabak et al., 2011; Sparbier et al., 2012; Ghebremedhin et al., 2016). Unfortunately, in the absence of automated analysis software, previous studies have used either manual calculations (Sparbier et al., 2012; Ghebremedhin et al., 2016) or self-developed algorithms with ambiguous cut-off values (Jung et al., 2014) to analyze the peak patterns. These techniques introduce intra- and inter-observer variability to the assay and are difficult to implement in routine diagnostic workflows. Recently, Bruker Daltonik launched a software module, the MALDI Biotyper™ Selective Testing for Beta-Lactamase Activity (MBT STAR-BL), for the automatic analysis of drug hydrolysis mass spectra. This module facilitates the simultaneous bacterial identification and detection of β-lactamase-mediated resistance toward ampicillin (AMP), piperacillin (PIP), cefotaxime (CTX), ceftazidime (CAZ), meropenem (MEM), and ertapenem (ETP).

The present study aimed to evaluate the ability of the MALDI Biotyper system equipped with the MBT STAR-BL module to identify bacteremia -causing bacteria and predict β-lactam resistance from plated isolates, as well as BC broths. The time-to-results determined using MBT STAR-BL were also compared with those obtained using a conventional culture-based method.

## Materials and methods

### Sample collection

In the first stage, 200 archived Gram-negative isolates of different species and various drug susceptibility patterns were collected and used to evaluate the ability of the MBT STAR-BL to detect β-lactamase-mediated resistance to all claimed antibiotics except ertapenem, which was not available locally. All strains were isolated from BCs previously collected at four different public hospitals throughout the territory. *Escherichia coli* strain DH5a was used as a β-lactamase-negative control strain, whereas ATCC *E. coli* strain BAA-2452, a NDM-1 carbapenemase producer, was used as a positive control in all drug hydrolysis tests.

In the second stage, 153 positive BC broths derived from patients with Gram-negative bacterial bloodstream infections were collected prospectively from January to December 2016. BACTEC™ FX (Becton Dickson, US) and BacT/Alert FA (bioMerieux, France) blood culture system are housed in hospitals. All positive BCs were subjected to direct Gram staining. If Gram-negative rods were found, a 5-mL aliquot of culture broth was transported to our laboratory for direct bacterial identification, followed by the detection of β-lactamase-mediated resistance using MBT STAR-BL within the same day.

On the following day, MALDI-TOF MS analyses were repeated using isolated colonies grown on subculture plates.

### Bacterial identification from BC broths using MALDI-TOF MS

For direct bacterial identification from BC broths, bacterial proteins were extracted using saponin-based protocol (Chen et al., 2013). The target plate was then analyzed using the Bruker Microflex LT system and MALDI Biotyper Compass software with the V5.0.0.0 spectra library (5989 spectra).

### Preparation of antibiotic solutions

Solutions of AMP (3 mg/mL), PIP (0.5 mg/mL), CTX (0.5 mg/mL), CAZ (0.25 mg/mL) and MEM (1 mg/mL) were prepared in incubation buffer (10 mM ammonium bicarbonate, 10 μg/mL zinc chloride, pH 8–9).

The antibiotics used for MBT STAR-BL measurement were selected according to the bacterial identification given by MALDI-TOF MS. If Enterobacteriaceae was identified, β-lactamase activity against AMP, CTX/CAZ and MEM were studied. For non-fermentative Gram-negative rods (NFGRs), including *Acinetobacter* spp. and *Pseudomonas* spp., β-lactamase activities against PIP, CAZ and MEM were investigated. β-lactamase activities against to all five β-lactam drugs was determined if no reliable ID was obtained.

### MBT-STAR-BL measurement

For plated isolates, sample preparation was done according to Hrabak et al. (2012). For BC broths, a bacterial pellet was isolated using saponin-based extraction method. Briefly, 1 mL of BC broth was treated with 5% saponin and subsequently washed twice with distilled water. After centrifugation, the bacterial pellet was resuspended in 50 μL of the appropriate antibiotic solution and incubated at 37°C under agitation (900 rpm) for 2 h, followed by centrifugation at 2,000 rpm for 2 min to collect the supernatant.

One microliter of supernatant was applied to the MSP96 target plate (reactions were performed in quadruplicate). The dried spots were overlaid with 1 μL of MBT STAR-BL Matrix. Automated mass spectrometric measurements were performed using the STAR-BL module (RUO version) embedded in the MALDI Biotyper Compass software. For instrument calibration, an antibiotic calibration standard (ACS; Bruker Daltonik) was measured in parallel with the samples in each run.

The STAR-BL module automatically calculated the normalized logRQ values for each sample. A value ≤0.2 indicated negative drug hydrolysis (i.e., reported as susceptible), whereas a value ≥0.4 indicated positive β-lactamase activity (i.e., reported as resistant). Normalized logQR values between 0.2 and 0.4 indicated an indeterminate hydrolysis measurement requiring retesting. If the repeated test yielded the same logQR value, the results were reported as indeterminate.

### Routine microbiological processing

Routine processing of BCs included the subcultivation of positive broths on Columbia Blood agar. The final identification involved a MALDI-TOF MS analysis of single isolated colonies grown on subculture plates.

Interim drug susceptibility patterns were determined by disk diffusion test directly using positive BC broths, whereas final drug susceptibilities relied on the disk diffusion testing patterns obtained from subcultured isolates (Clinical and Laboratory Standards Institute, 2013). Furthermore, the extended-spectrum β-lactamase (ESBL) and carbapenemase phenotypes were confirmed using a combined disk method (Clinical and Laboratory Standards Institute, 2013; Pournaras et al., 2013).

### Strain characterization

The presence of plasmid-mediated β-lactamases in all identified β-lactam-resistant Enterobacteriaceae was confirmed using multiplex PCR assays, followed by amplicon sequencing-based genotyping (Dallenne et al., 2010; Doyle et al., 2012). PCR sequencing was used to detect the mutations in the *ampC* promoter/attenuator region as well as in *ampD and ampR* which are associated with hyperproduction of AmpC in Enterobacteriaceae (Kaneko et al., 2005; Schmidtke and Hanson, 2006; Peter-Getzlaff et al., 2011). For NFGRs, carbapenemases were characterized using a dual-tube multiplex PCR (Kazi et al., 2015), whereas some rare β-lactamases, such as *bla*_*Pom*_ and *bla*_*L*1_, were detected as previously described (Thaller et al., 2011; Yang et al., 2014). For cases involving discrepancy between the phenotypic DST and MBT STAR-BL assays, the minimal inhibitory concentration (MIC) was determined using an *E*-test (bioMérieux, Marcy l'Etoile, France) according to the manufacturer's guidelines.

### Assessment of the times-to-results using the MBT STAR-BL and routine culture methods

**Figure 1:**
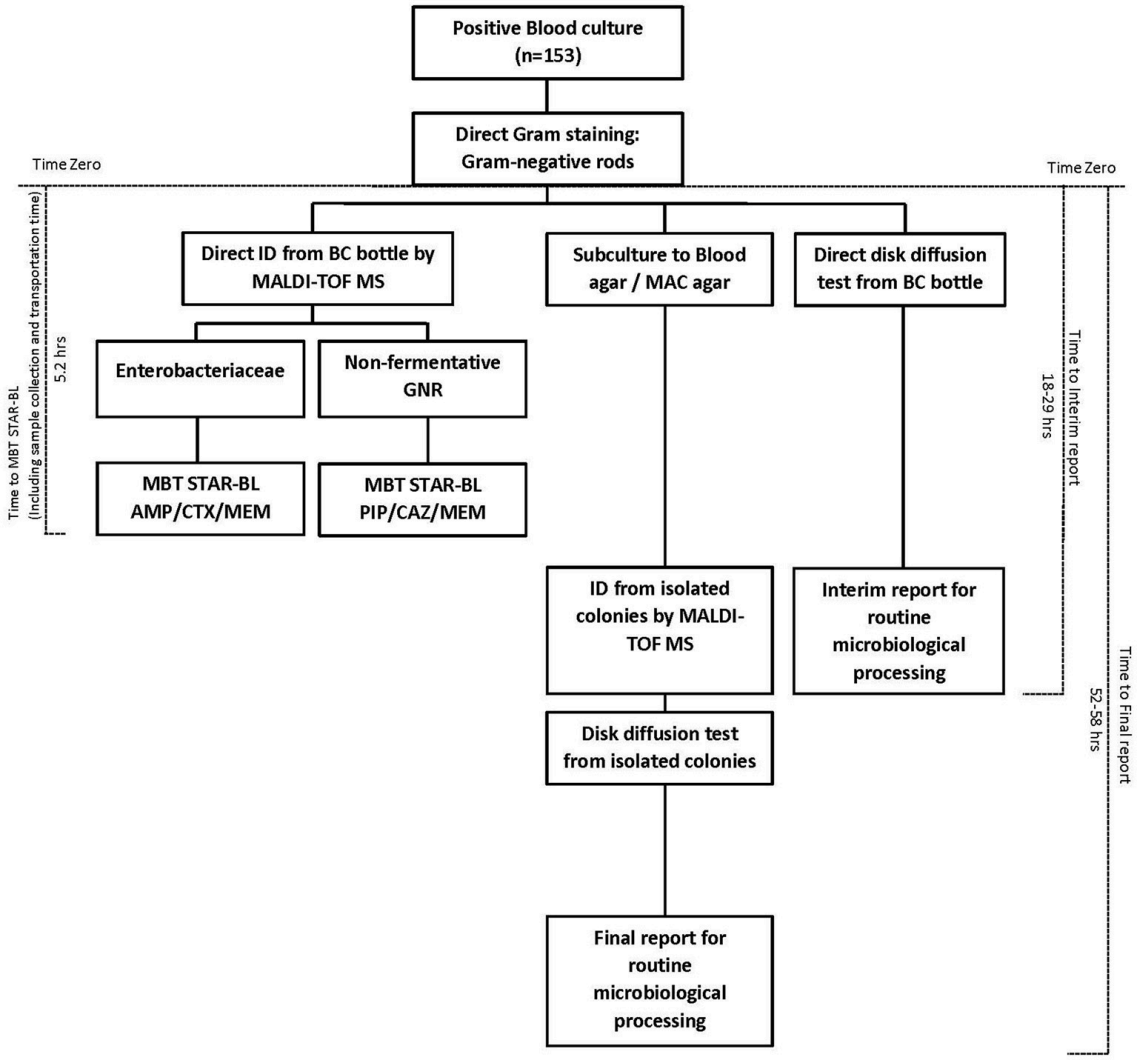
Diagram of the workflow and turnaround time of bacterial identification and β-lactam resistance detection from positive blood cultures, using MBT STAR-BL and routine microbiological processing.

The times-to-results were compared based on a subset of 153 monomicrobial BCs (*n* = 88) collected from a hospital adjacent to our laboratory that houses the MALDI Biotyper system with STAR-BL module. BCs that were identified as positive between 7:00 a.m. and 4:00 p.m. were collected at the hospital. MBT STAR-BL measurements were performed immediately upon sample arrival in our laboratory. Time-zero was defined as the time at which the primary Gram staining result was reported. For MALDI-TOF MS analysis, the time elapsed between time-zero and the MBT STAR-BL analysis completion time was considered the “Time-to-MBT STAR-BL.” For routine microbiological processing, the total time required to obtain bacterial identification and the results of the direct (interim) disk diffusion test was defined as the “Time-to-interim report,” whereas the total time required to obtain the final drug susceptibility result from subcultured isolates was defined as the “Time-to-final report” (Figure 1).

## Results

### MBT STAR-BL testing on archived cultured isolates

Among 139 Enterobacteriaceae collected in this study, 103 were AMP-resistant, as determined by phenotypic DST, and 94 (91.3%) exhibited positive hydrolysis in the MBT STAR-BL module. Notably, 63/66 (95.5%) AMP-resistant *E. coli* isolates were correctly identified by MBT STAR-BL with 100% specificity (Table 1).

**Table 1:** Performance of the MBT STAR-BL module for the detection of β-lactamase-mediated resistance in cultured isolates.

**Bacterial species and β-lactamase genes**	**No. of isolates**	**No. of resistant isolates confirmed by disk diffusion test**				**Positive hydrolysis detected by MBT STAR BL^b^**			
		**AMP/PIP^a^**	**CTX**	**CAZ**	**MEM**	**AMP/PIP**	**CTX**	**CAZ**	**MEM**
***Escherichia coli***									
None	35	0	0	0	0	0	0	0	0
TEM-1b/1c	28	28	0	0	0	28	0	0	0
CTX-M-13 (ESBL)	7	7	7	2	0	7	7	1	0
CTX-M-14 (ESBL)	13	13	13	5	0	13	13	1	0
TEM-72 (ESBL	1	1	1	0	0	1	1	0	0
TEM-1b + OXA-15 (ESBL)	3	3	3	0	0	3	3	0	0
Hyper AmpC^c^	4	4	2	2	0	1	1	0	0
CMY-2 AmpC	1	1	1	1	0	1	1	0	0
NDM-5	6	6	6	6	6	6	6	6	6
KPC-2	3	3	3	3	3	3	3	3	3
***Klebsiella pneumoniae***									
SHV-1/11	16	16	0	0	0	13	0	0	0
SHV-1 + CTX-M-24 (ESBL)	1	1	1	0	0	1	1	0	0
SHV-1 + OXA (ESBL)	1	1	1	1	0	1	1	1	0
SHV-1 + DHA-1 AmpC	1	1	1	1	0	1	1	1	0
SHV-1 + NDM-1	1	1	1	1	1	1	1	1	1
***Klebsiella oxytoca***									
SHV-1 + IMP-8	3	3	3	3	3	3	3	3	3
***Proteus mirabilis***									
TEM-1b	6	6	0	0	0	6	0	0	0
***Salmonella*** **spp**.									
None	1	0	0	0	0	0	0	0	0
***Citrobacter freundii***									
Inducible AmpC + NDM-1	1	1	1	1	1	1	1	1	1
***Enterobacter cloacae***									
Inducible AmpC^d^	3	3	0	0	0	0	0	0	0
Inducible AmpC + CMY-2 AmpC	2	2	2	2	0	2	2	2	0
Inducible AmpC + IMP-1	2	2	2	2	2	2	2	2	2
Total *Enterobacteriaecae*	139	103	48	30	16	94	47	22	16
***Pseudomonas aeruginosa***									
Inducible AmpC	30	0	ND	0	0	0	ND	0	0
Inducible AmpC + VIM-4	3	3	ND	3	3	3	ND	3	3
***Acinetobacter baumannii***									
OXA-23, −51	28	28	ND	20	28	28	ND	0	28
Total NFGR	61	31	ND	23	31	31	ND	3	31
Total organisms	200	134	48	53	47	125	47	25	47

a *An AMP hydrolysis assay was applied to Enterobacteriaceae isolates, whereas a PIP hydrolysis assay was used for all NFGRs, including Pseudomonas spp. and Acinetobacter spp*.

b *Isolates were considered resistant only when the logRQ value was ≥0.4 in the MBT STAR-BL module*.

c *Hyper AmpC refers to E. coli strains with insertions of 1 or 2 bases between the 35 and 10 boxes of the ampC promoter region, which were shown to cause ampC hyperexpression (Peter-Getzlaff et al., 2011)*.

d *Inducible AmpC refers to bacterial species that harbored an inducible chromosomal ampC gene with intact regulatory elements (i.e., no ampD and ampR mutations)*.

MBT STAR-BL successfully identified 47/48 (97.9%) CTX-resistant and 22/30 (73.3%) CAZ-resistant Enterobacteriaceae isolates with no false positivity (Table 1). Interestingly, all 26 ESBL-producing Enterobacteriaceae demonstrated positive CTX hydrolysis, whereas only 3 (11.5%) could hydrolyze CAZ.

Remarkably, all carbapenemase-producing Enterobacteriaceae (*n* = 16) isolates were correctly identified by MBT STAR-BL with 100% specificity (Table 1).

For NFGRs, MBT STAR-BL successfully detected PIP and MEM hydrolysis in all *A. baumannii* isolates harboring *bla*_*OXA*−23_ and *bla*_*OXA*−51_ (*n* = 28) and all *P. aeruginosa* isolates harboring *bla*_*VIM*_ (*n* = 3) (Table 1). However, all *A. baumannii* isolates harboring *bla*_*OXA*−23_ and *bla*_*OXA*−51_ failed to exhibit CAZ hydrolysis in MBT-STAR-BL.

The characteristics of the bacterial cultures with discrepant results are shown in Table **4**.

### MBT STAR-BL testing on monomicrobial blood culture broths

A total of 153 positive BC bottles, including monomicrobial (*n* = 134) and polymicrobial cultures (*n* = 19), were prospectively collected for this study.

Direct MALDI-TOF MS correctly identified bacteria in 116/134 (86.6%) monomicrobial BCs, and achieved species-level identification in 87/134 (64.9%) (Table S1).

Among 112 monomicrobial BCs harboring AMP-resistant Enterobacteriaceae, 90 (80.4%) exhibited AMP hydrolysis in MBT STAR-BL (Table 2). False negative results were mainly obtained from species that harbored inducible chromosomal *ampC* (Table **4**). For *E. coli*, 58/63 (92.1%) AMP-resistant cultures were correctly identified by MBT STAR-BL, with the specificity of 92.9% (Table **5**).

**Table 2:** Performance of the MBT STAR-BL module in the detection of β-lactamase-mediated resistance in prospectively collected monomicrobial blood cultures.

**Bacterial Species and β-lactamase genes**	**No. of cultures**	**No. of resistant isolates confirmed by disk diffusion test**			**True and (false) positive hydrolysis detected from blood culture broths by MBT STAR BL**			**True and (false) positive hydrolysis detected from subcultured isolates by MBT STAR BL**		
		**AMP/PIP^a^**	**CTX/CAZ^b^**	**MEM**	**AMP/PIP^a^**	**CTX/CAZ^b^**	**MEM**	**AMP/PIP^a^**	**CTX/CAZ^b^**	**MEM**
***Escherichia coli***										
None	14	0	0	0	0 (1)	0 (1)	0 (1)	0	0	0
TEM-1b/1c	38	38	0	0	35	0 (2)	0	37	0	0
CTX-M-13 (ESBL)	10	10	10	0	8	6	0 (2)	10	10	0
TEM-1b + CTX-M-13 (ESBL)	4	4	4	0	4	3	0	4	4	0
TEM-1b + CTX-M-14 (ESBL)	7	7	7	0	7	5	0	7	7	0
SHV-1 + CTX-M-39 (ESBL)	1	1	1	0	1	1	0	1	1	0
CMY-2 AmpC	2	2	2	0	2	0	0	2	1	0
DHA-1 AmpC	1	1	1	0	1	1	0	1	1	0
***Klebsiella pneumoniae***										
SHV-1	27	27	0	0	17	0 (4)	0	22	0	0
SHV-1 + CTX-M-14 (ESBL)	4	4	4	0	4	3	0	4	4	0
DHA-1 AmpC	1	1	1	0	1	1	0	1	1	0
***Klebsiella oxytoca***										
SHV-1	1	1	0	0	1	0	0	1	0	0
***Proteus mirabilis***										
TEM-1b/1c	3	3	0	0	2	0	0	3	0	0
TEM-1b + CTX-M-14 (ESBL)	2	2	2	0	2	2	0	2	2	0
***Salmonella*** **spp**.										
TEM-1b	1	1	0	0	1	0	0	1	0	0
***Morganella morganii***										
Inducible AmpC^d^	2	2	0	0	1	0	0	1	0	0
***Enterobacter cloacae***										
Inducible AmpC^d^	2	2	0	0	0	0	0	0	0	0
***Citrobacter freundii***										
Inducible AmpC^d^	3	3	0	0	3	0 (1)	0	3	0	0
***Pluralibacter gergoviae***										
Inducible AmpC^d^	1	1	0	0	0	0	0	0	0	0
***Raoultella ornithinolytica***										
Inducible AmpC^d^	2	2	0	0	0	0	0	2^c^	0	0
Total Enterobacteriaceae	126	112	32	0	90 (1)	22 (8)	0 (3)	102	31	0
***Acinetobacter baumannii***										
None	2	0	0	0	0	0	0	0	0	0
OXA-23, −51	2	2	2	2	2	0	0	2	0	2
***Pseudomonas aeruginosa***										
Inducible AmpC^d^	1	0	0	0	0	0	0	0	0	0
***Pseudomonas otitidis***										
POM-1	1	0	0	1	0	0	0	0	0	1
***Stenotrophomonas maltophilia***										
MBL L1	2	2	2	2	2	0	2	2	0	2
Total NFGRs	8	4	4	5	4	0	2	4	0	5
Total organisms	134	116	36	5	94 (1)	22 (8)	2 (3)	106	31	4

a *An AMP hydrolysis assay was applied to Enterobacteriaceae, whereas a PIP hydrolysis assay was used for all NFGRs, including Pseudomonas spp. and Acinetobacter spp*.

b *A CTX hydrolysis assay was applied to Enterobacteriaceae, whereas a CAZ hydrolysis assay was used for all NFGRs*.

c *The subcultured isolates of R. ornithinolytica failed to hydrolyze AMP after a 2-h incubation. However, the hydrolysis became positive when the incubation time was extended to 4 h*.

d *Inducible AmpC refers to bacterial species that harbored an inducible chromosomal ampC gene with intact regulatory elements (i.e., no ampD and ampR mutations)*.

In addition, MBT STAR-BL successfully detected CTX resistant Enterobacteriaceae in 22/32 (68.8%) BCs (Table 2). Notably, of 28 ESBL-producing Enterobacteriaceae, 20 (71.4%) demonstrated positive CTX hydrolysis (Table 2). The false negative MBT STAR-BL results did not correlate with CTX-M genotypes or MIC levels (Table **4**). Moreover, eight BCs containing CTX-susceptible Enterobacteriaceae yielded false positive hydrolysis results, resulting in a specificity of 91.5% (Table **5**).

Unfortunately, no carbapenem-resistant Enterobacteriaceae were collected from BCs in this study.

For NFGRs, four BCs were resistant to PIP and CAZ as determined by phenotypic DST. MBT STAR-BL correctly predicted PIP resistance for all of them, but none of the BCs showed detectable CAZ hydrolysis in MBT STAR-BL. In addition, among 5 NFGRs that haboured carbapenemase genes and exhibited MEM resistance in phenotypic DST, MBT STAR-BL only detected MEM hydrolysis in 2 (40%) of the BCs (Table 2).

The characteristics of the BCs with discrepant results are shown in Table **4**.

MALDI-TOF MS analyses were repeated for each sample using plated isolates subcultivated from BCs on the following day. The diagnostic sensitivities and specificities of MBT STAR-BL for the subcultured plates resembled those obtained from the archived cultured isolates (Tables 2, **5**).

### MBT STAR-BL testing on polymicrobial blood culture broths

Direct MALDI-TOF MS correctly identified at least 1 bacterial species in 16/19 (84.2%) polymicrobial BCs (Table 3).

**Table 3:** Performance of the MALDI-TOF MS workflow for bacterial identification and β-lactamase-mediated resistance detection in polymicrobial blood cultures and respective subcultured isolates.

**Blood culture broths**	**Conventional ID and DST**							**MALDI-TOF MS ID and MBT STAR-BL from blood culture broths**				**MALDI-TOF MS ID and MBT STAR-BL from subcultured isolates**			
	**ID**	**β-lactamase**	**AMP/PIP**	**CAZ**	**CTX**	**MEM**	**ESBL**	**ID**	**AMP/PIP**	**CTX/CAZ**	**MEM**	**ID**	**AMP/PIP**	**CTX/CAZ**	**MEM**
BC6	*E. cloacae*	Inducible AmpC^a^	R	S	S	S	–	*Enterobacter sp*.	*I^c^*	*S^c^*	S	*E. cloacae*	*S^c^*	*S^c^*	S
	*E. cloacae*	Derepressed AmpC^b^	R	R	R	S	–					*E. cloacae*	*I^c^*	*S^c^*	S
BC11	*E. coli*	CTX-M-13	R	S	R	S	+	*E. coli*	R	R	S	*E. coli*	R	R	S
	*E. coli*	TEM-1c	R	S	S	S	–					*E. coli*	R	S	S
BC23	*K. pneumoniae*	SHV-1	R	S	S	S	–	No reliable ID	R	S	S	*K. pneumoniae*	R	S	S
	*E. cloacae*	Inducible AmpC	R	S	S	S	–					*E. cloacae*	*S^c^*	S	S
BC26	*E. coli*	CTX-M-13	R	R	R	S	+	*E. coli*	R	R	S	*E. coli*	R	R	S
	*P. mirabilis*	TEM-1b	R	S	S	S	–					*P. mirabilis*	R	S	S
BC27	*A. baumannii*	OXA-23,−45	R	R	ND	R		*A. baumannii*	R	*S^c^*	R	*A. baumannii*	R	*S^c^*	R
	*A. baumannii*	OXA-23,−45	R	R	ND	R						*A. baumannii*	R	*S^c^*	R
	*P. aeruginosa*	Inducible AmpC	S	S	ND	S						*P. aeruginosa*	S	S	S
BC30	*K. pneumoniae*	SHV-1	R	S	S	S	–	*E. coli*	R	S	S	*K. pneumoniae*	R	S	S
	*E. coli*	TEM-1b	R	S	S	S	–					*E. coli*	R	S	S
BC33	*K. pneumoniae*	TEM-1b, SHV-1	R	S	S	S	–	*Klebsiella sp*.	R	R	S	*K. pneumoniae*	R	S	*S*
	*K. pneumoniae*	CTX-M-39	R	R	R	S	+					*K. pneumoniae*	R	R	*S*
BC37	*P. aeruginosa*	Inducible AmpC	S	S	ND	S		*Pseudomonas sp*.	S	S	S	*P. aeruginosa*	S	S	*S*
	*M. morganii*	Inducible AmpC	R	S	S	S	–					*M. morganii*	*S^c^*	S	S
BC43	*M. morganii*	Inducible AmpC	R	S	S	S	–	*M. morganii*	*I^c^*	S	S	*M. morganii*	*S^c^*	S	S
	*P. aeruginosa*	Inducible AmpC	S	S	ND	S	–					*P. aeruginosa*	S	S	S
BC54	*K. pneumoniae*	SHV-1	R	S	S	S	–	No reliable ID	S	S	S	*K. pneumoniae*	R	S	I
	*K. pneumoniae*	SHV-1	R	S	S	S	–					*K. pneumoniae*	R	S	I
BC55	*E. coli*	CTX-M-14	R	S	R	S	+	*E. coli*	R	*S^c^*	S	*E. coli*	R	R	S
	*E. coli*	None	S	S	S	S						*E. coli*	S	S	S
BC67	*E. coli*	None	I	S	S	S	–	*E. coli*	R	S	S	*E. coli*	S	S	S
	*E. coli*	TEM-1b	R	S	S	S	–					*E. coli*	*I^c^*	S	S
BC72	*E. coli*	TEM-1b, CTX-M-13	R	S	R	S	+	*E. coli*	R	R	S	*E. coli*	R	R	S
	*E. coli*	CTX-M-9	R	S	R	S	+					*E. coli*	R	R	S
BC75	*K. pneumoniae*	SHV-1	R	S	S	S	–	No reliable ID	R	I	S	*K. pneumoniae*	R	S	I
	*K. oxytoca*	SHV-1	R	S	S	S	–					*K. oxytoca*	R	S	I
BC83	*E. coli*	TEM-1c	R	S	S	S	–	*E. coli*	R	S	S	*E. coli*	R	I	S
	*E. coli*	None	I	S	S	S	–					*E. coli*	S	I	S
BC86	*E. coli*	CTX-M-24	R	S	R	S	+	*E. coli*	R	*I^c^*	S	*E. coli*	I	R	S
	*E. coli*	OXA-15	R	S	R	S	+					*E. coli*	I	R	S
BC110	*E. coli*	TEM-1b	R	S	S	S	–	*E. coli*	R	S	I	*E. coli*	R	S	S
	*E. coli*	OXA-1	R	S	S	S	–					*E. coli*	R	S	S
PY21	*E. coli*	OXA-1, CTX-M-13 & CTX-M-37	R	R	R	S	+	*E. coli*	R	R	S	*E. coli*	R	R	S
	*K. pneumoniae*	SHV-1	R	S	S	S	–					*K. pneumoniae*	R	S	S
PY37	*E. coli*	None	S	S	S	S		*E. coli*	S	S	S	*E. coli*	S	S	S
	*Plesiomonas Shigelloides*	None	S	S	S	S	–					*Plesiomonas Shigelloides*	S	S	S

a *Inducible AmpC refers to bacterial species that harbored an inducible chromosomal ampC gene with intact regulatory elements (i.e., no ampD and ampR mutations)*.

b *The E. cloacae strain was found to harbor a truncated ampD gene, which was shown to fully derepress AmpC activity (Schmidtke and Hanson, 2006)*.

c *The underlined results indicate mismatches between conventional DST and MBT STAR-BL*.

*ND, Not done; NA, Not applicable*.

Additionally, MBT STAR-BL successfully detected AMP hydrolysis in all 10 BCs containing AMP-resistant *E. coli* strains. Among the 7 polymicrobial BCs containing ESBL-producing Enterobacteriaceae, 5 (71.4%) exhibited CTX hydrolysis in an MBT STAR-BL test (Table 3).

Full concordance was yielded between the MBT STAR-BL and phenotypic DST in prediction of PIP resistance in polymicrobial BCs containing NFGRs (Table 3). For the BCs containing two *A. baumannii* strains harboring *bla*_*OXA*−23__&51_, MBT STAR-BL successfully identified MEM hydrolysis in both the BC broths and subcultured isolates (Table 3). Overall, the β-lactam resistance patterns predicted by MBT STAR-BL were concordant with the phenotypic DST in 14/19 (73.7%) polymicrobial BCs. Details of the mismatches are shown in Table 4.

**Table 4:** The characteristics of bacterial strains with discrepant results from the phenotypic drug susceptibility test and MBT STAR-BL analysis.

**Bacterial Species**	**β-lactamase genes**	**No. of strains**	**Source**	**MIC range (μg/ml), S/R**	**MBT STAR-BL from blood culture**		**MBT STAR-BL from isolates**	
					**logRQ value range**	**Results**	**logRQ value range**	**Results**
**MISMATCHES FOR AMP SUSCEPTIBILITY**								
*E. coli*	Hyperexpressed AmpC^b^	3	Retrospective isolates	64 to >256, R	NA	NA	0.21 to 0.29	I
*E. coli*	TEM-1b/1c	3	Monomicrobial BC	>256, R	−0.23 to 0.21	S/I	0.27 to 0.47	I/R
*E. coli*	CTX-M-13	2	Monomicrobial BC	>256, R	0.14 to 0.24	S/I	0.82 to 1.12	R
*K. pneumoniae*	SHV-1	3	Retrospective isolates	>256, R	NA	NA	−0.1 to 0.23	S/I
*K. pneumoniae*	SHV-1	10	Monomicrobial BC	128 to >256, R	−0.41 to 0.27	S/I	−0.3 to 1.17	S/I/R
*K. pneumoniae*	SHV-1	2	Polymicrobial BC	>256, R	−0.34	S	1.02 to 1.15	R
*P. mirabilis*	TEM-1c	1	Monomicrobial BC	128, R	0.02	S	0.34	I
*E. cloacae*	Inducible AmpC	3	Retrospective isolates	128 to 256, R	NA	NA	−0.24 to 0.28	S/I
*E. cloacae*	Inducible AmpC	2	Monomicrobial BC	128, R	−0.11 to 0.15	S	−0.3 to 0.37	S
*E. cloacae*	Inducible AmpC	1	Polymicrobial BC	128, R	0.29	I	−0.31	S
*E. cloacae*	Derepressed AmpC^c^	1	Polymicrobial BC	256, R	0.29	I	0.25	I
*M. morganii*	Inducible AmpC	1	Monomicrobial BC	256, R	−0.16	S	0.24	I
*M. morganii*	Inducible AmpC	2	Polymicrobial BC	128 to 256, R	−0.43 to 0.23	S/I	−0.52 to −0.44	S
*P. gergoviae*	Inducible AmpC	1	Monomicrobial BC	256, R	−0.35	S	0.32	I
*R. ornithinolytica*	Inducible AmpC	2	Monomicrobial BC	128 to 256, R	−0.24 to 0.1	S	0.4 to 1.33	R^a^
**MISMATCHES FOR CTX SUSCEPTIBILITY**								
*E. coli*	Hyperexpressed AmpC^b^	1	Retrospective isolates	>16, R	NA	NA	0.15	S
*E. coli*	CTX-M-13	4	Monomicrobial BC	>16, R	−0.13 to 0.36	S/I	0.44 to 0.96	R
*E. coli*	TEM-1b + CTX-M-13	1	Monomicrobial BC	>16, R	0.21	I	0.51	R
*E. coli*	TEM-1b + CTX-M-14	2	Monomicrobial BC	>16, R	0.18 to 0.31	S/I	0.56 to 0.67	R
*E. coli*	CTX-M-14	1	Polymicrobial BC	>16, R	0.13	S	0.67	R
*E. coli*	CTX-M-24	1	Polymicrobial BC	>16, R	0.21	I	1.04	R
*E. coli*	CMY-2 AmpC	2	Monomicrobial BC	>16, R	0.10 to 0.20	S/I	−0.03 to 0.56	S/R
*K. pneumoniae*	SHV-1 + CTX-M-14	1	Monomicrobial BC	>16, R	0.12	S	0.52	R
*E. cloacae*	Derepressed AmpC^c^	1	Polymicrobial BC	>16, R	0.33	I	0.28	I
**MISMATCHES FOR CAZ SUSCEPTIBILITY**								
*E. coli*	CTX-M-13	1	Retrospective isolates	16 to >256, R	NA	NA	−0.32 to −0.04	S
*E. coli*	CTX-M-14	4	Retrospective isolates	16 to >256, R	NA	NA	−0.23 to 0.08	S
*E. coli*	Hyperexpressed AmpC^b^	2	Retrospective isolates	32, R	NA	NA	−0.03 to 0.15	S
*E. coli*	CMY-2 AmpC	1	Retrospective isolates	128, R	NA	NA	−0.13	S
*A. baumannii*	OXA-23, −51	28	Retrospective isolates	>256, R	NA	NA	−0.43 to 0.16	S
*A. baumannii*	OXA-23, −51	2	Monomicrobial BC	>256, R	−0.45 to −0.55	S	−0.53 to −0.18	S
*A. baumannii*	OXA-23, −51	2	Polymicrobial BC	>256, R	−0.95	S	−0.17	S
*S. maltophila*	MBL L1	2	Monomicrobial BC	>256, R	−1.63 to −0.25	S	−2.28 to −0.13	S
**MISMATCHES FOR MEM SUSCEPTIBILITY**								
*A. baumannii*	OXA-23, −51	2	Monomicrobial BC	>32, R	0.21–0.25	I	0.58–0.77	R
*P. otitidis*	POM-1	1	Monomicrobial BC	4, R	0.32	I	0.83	R

a *The subculture isolates of R. ornithinolytica failed to hydrolyze AMP after a 2-h incubation. However, the hydrolysis became positive when the incubation time was extended to 4 h*.

b *Hyper AmpC refers to E. coli strains that harbored insertions of 1 or 2 bases between the 35 and 10 boxes in the ampC promoter region, which were shown to cause ampC hyperexpression (Peter-Getzlaff et al., 2011)*.

c *The E. cloacae strain was found to harbor a truncated ampD gene, which was previously shown to fully derepress AmpC activity (Schmidtke and Hanson, 2006)*.

**Table 5:** The overall diagnostic performances of drug hydrolysis assays for cultured isolates and blood cultures at logRQ cut-off values of 0.4 and 0.2.

	**Drug hydrolysis assay**											
	**AMP^a^**		**AMP (For** ***E.coli*** **only)**		**PIP^b^**		**CTX^c^**		**CAZ^d^**		**MEM^e^**	
	**Sn %**	**Sp (%)**	**Sn (%)**	**Sp (%)**	**Sn (%)**	**Sp (%)**	**Sn (%)**	**Sp (%)**	**Sn (%)**	**Sp (%)**	**Sn (%)**	**Sp (%)**
**RETROSPECTIVE ISOLATES**												
logRQ cut-off = 0.4	91.3 (94/103)	100 (36/36)	95.5 (63/66)	100 (35/35)	100 (31/31)	100 (30/30)	97.9 (47/48)	100 (91/91)	47.2 (25/53)	100 (147/147)	100 (47/47)	100 (153/153)
logRQ cut-off = 0.2	96.1 (99/103)	97.2 (35/36)	100 (66/66)	100 (35/35)	100 (31/31)	100 (30/30)	97.9 (47/48)	100 (91/91)	47.2 (25/53)	100 (147/147)	100 (47/47)	100 (153/153)
**BLOOD CULTURE BOTTLES**												
logRQ cut-off = 0.4	80.4 (90/112)	92.9 (13/14)	92.1 (58/63)	92.9 (13/14)	100 (4/4)	100 (4/4)	68.8 (22/32)	91.5 (86/94)	0 (0/4)	100 (4/4)	40 (2/5)	97.7 (126/129)
logRQ cut-off = 0.2	85.7 (96/112)	92.9 (13/14)	95.2 (60/63)	92.9 (13/14)	100 (4/4)	100 (4/4)	87.5 (28/32)	89.4 (84/94)	0 (0/4)	100 (4/4)	100 (5/5)	95.3 (123/129)
**SUBCULTURED ISOLATES FROM BC**												
logRQ cut-off = 0.4	91.1 (102/112)	100 (14/14)	98.4 (62/63)	100 (14/14)	100 (4/4)	100 (4/4)	96.9 (31/32)	100 (94/94)	0 (0/4)	100 (4/4)	100 (5/5)	100 (129/129)
logRQ cut-off = 0.2	96.4 (108/112)	85.7 (12/14)	100 (63/63)	85.7 (12/14)	100 (4/4)	100 (4/4)	96.9 (31/32)	93.6 (88/94)	0 (0/4)	100 (4/4)	100 (5/5)	97.7 (126/129)

a *In this column, the diagnostic performance of the AMP hydrolysis assay includes all Enterobacteriaceae*.

b *The PIP hydrolysis assay was only applied to NFGRs*.

c *The CTX hydrolysis assay was only applied to Enterobacteriaceae*.

d *In the first part of the study (retrospective isolates), a CAZ hydrolysis assay was applied to both Enterobacteriaceae and NFGR isolates, whereas in the second part of the study (blood culture), only NFGRs were included in the CAZ hydrolysis assay*.

e *The MEM hydrolysis assay were used for both Enterobacteriaceae and NFGR in both the cultured isolate or blood culture stages of the study*.

### Assessment of the times-to-results using MBT STAR-BL and routine microbiological processing

The times-to-results based on MBT STAR-BL and routine microbiological processing were investigated for 88 monomicrobial BCs (Table 6). Among the BCs, the average time to MBT STAR-BL was 5.2 h. For Enterobacteriaceae, the MALDI-TOF MS-based workflow allowed the laboratory to identify bacterial species and β-lactamase-mediated resistance to β-lactams at averages of 13.5 and 47.5 h before the interim and final reporting of the routine processing, respectively. For NFGRs, the MBT STAR-BL assay enabled the reporting of bacterial identification and MEM hydrolysis at 23.0 and 49.9 h before the interim and final routine processing, respectively.

**Table 6:** Assessment of the times to results, based on MBT STAR-BL and routine microbiological processing.

**Organisms**	**No. of isolates^a^**	**Average time to MBT STAR-BL (h)^b^**	**Time to interim report by routine microbiological processing (h)^c^**			**Time to final report by routine microbiological processing (h)^d^**		
			**Average (h)**	**Δ Time to Result^e^**	***p*-value**	**Average (h)**	**Δ Time to Result^e^**	***p*-value**
Enterobacteriaceae	82	5.2	18.72	13.52	<0.0001	52.68	47.48	<0.0001
ESBL producers	21	5.2	18.97	13.77	<0.0001	54.25	49.05	<0.0001
Non-ESBL producers	61	5.2	18.02	12.82	<0.0001	48.15	42.95	<0.0001
NFGR	6	5.2	28.17	22.97	<0.0001	55.08	49.88	<0.0001
*Pseudomonas spp*.	3	5.2	27.33	22.13	<0.0001	52	46.8	<0.0001
*A. baumannii*	3	5.2	29	23.8	<0.0001	58.17	52.97	<0.0001
All organisms	88	5.2	19.36	14.16	<0.0001	52.84	47.64	<0.0001

a *Only monomicrobial BCs with concordant results between the MBT STAR-BL and routine microbiological processing were included in the time to results assessment*.

b *The time to MBT STAR-BL refers to the time elapsed between the reporting of the primary Gram stain result and the completion of the MBT STAR-BL analysis (including the pending time before collection, transportation time, and times used for protein extraction, antibiotic incubation and MADLI-TOF MS measurement)*.

c *The time to interim report refers to the time required to obtain bacterial identification and the results of a direct disk diffusion test of inoculates of BC broths in a clinical microbiology laboratory*.

d *The time to final report refers to the time required to obtain bacterial identification and the results of the final disk diffusion test of inoculates from colonies isolated from subcultured plates in a clinical microbiology laboratory*.

e *This column indicates the differences in the times to results between MBT STAR-BL and the interim and final reports of routine microbiological processing*.

## Discussion

In this study, we evaluated the practicality of MALDI-TOF MS using the 5989 spectra library, followed by MBT STAR-BL as a diagnostic workflow, for the identification of bacterial species and β-lactamase-mediated resistance in cultured isolates and patient-derived BCs. In contrast to previous studies that mainly investigated origin of carbapenem resistance in Enterobacteriaceae (Hrabak et al., 2011, 2012; Papagiannitsis et al., 2015; Ghebremedhin et al., 2016; Monteferrante et al., 2016; Oviano et al., 2017), this study identified the susceptibilities of a wide spectrum of bacterial species harboring different enzymes. To the best of our knowledge, this is the most comprehensive evaluation of the MBT STAR-BL module since its official launch in 2016.

**Figure 2:**
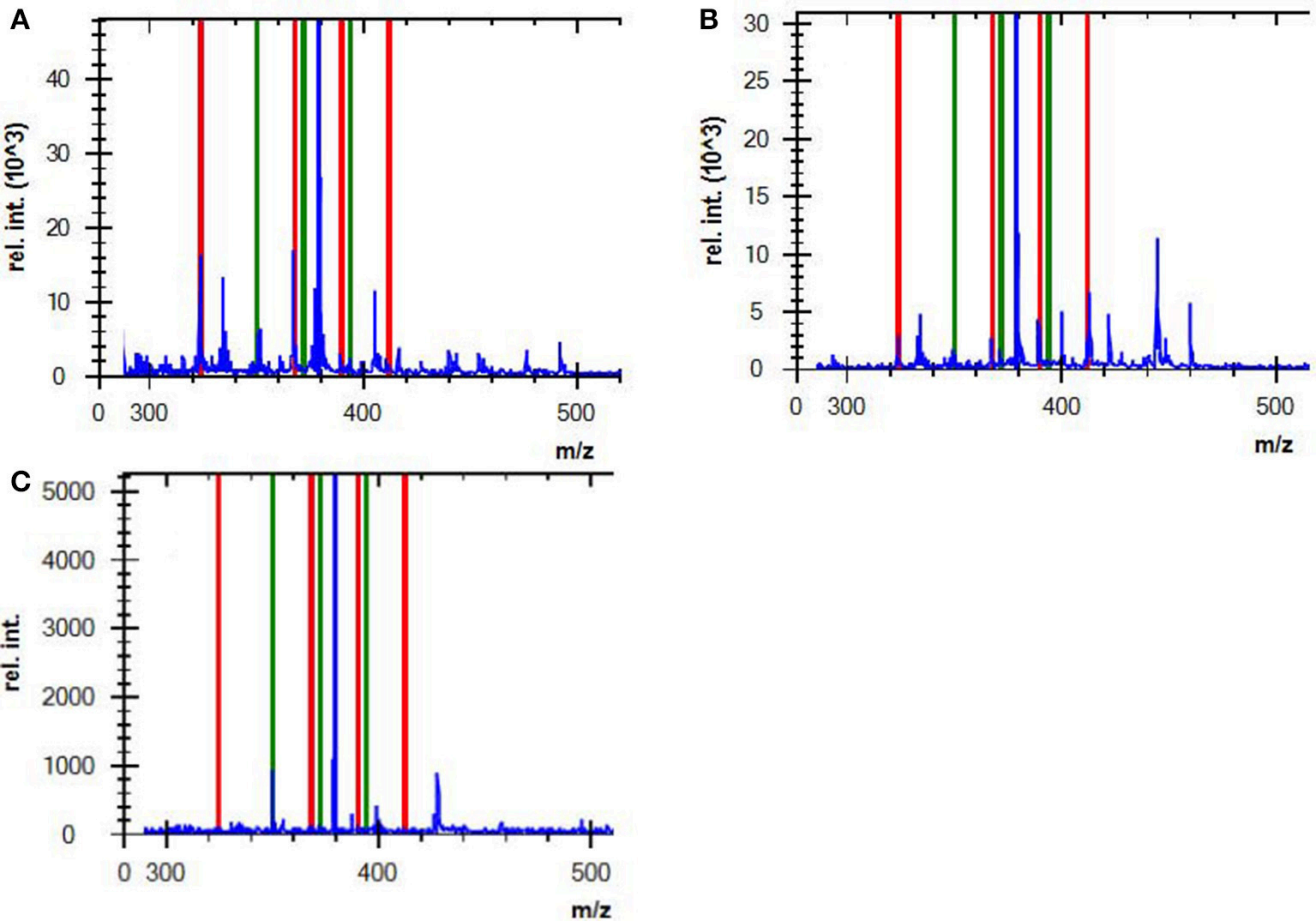
Mass spectra of **(A)** a complete AMP hydrolysis obtained from a positive control strain (ATCC BAA-2452, NDM-1-producing *E. coli*) incubated with AMP for 2 h, **(B)** an incomplete AMP hydrolysis obtained from an *E. coli* strain that hyperexpressed *ampC*, and **(C)** non-hydrolyzed AMP from a negative control strain (DH5α). Red lines indicate the hydrolyzed antibiotic peaks, and green lines indicate intact antibiotic peaks (non-hydrolyzed).

AMP is used as a surrogate marker of resistance to aminopenicillins in *E. coli* and other Enterobacteriaceae, such as *Proteus mirabilis, Salmonella* spp*. and Shigella* spp. In this study, AMP resistance was detected in *E.coli* from cultured isolates and BCs at detection sensitivities of 95.5 and 92.1%, respectively (Table 5), consistent with Jung et al. (2014). Although we did not observe a correlation between the logRQ values and MIC levels, we noted that all the strains with high MIC level but low logRQ value are those harboring mutations associated with hyperproduction of AmpC (Table 4). An analysis of the respective mass spectra revealed that the peaks corresponding to hydrolyzed AMP were weak and coexistent with the molecular peaks of intact antibiotics, indicating incomplete drug hydrolysis (Figure 2). We repeated this assay for all chromosomal *ampC* carriers after extending the incubation time to 4 h. Unfortunately, only two *R. ornithinolytica* isolates yielded logRQ values ≥ 0.4 whereas most others had values of 0.2–0.4.

Both CTX and CAZ could be used as indicator drugs for the MBT STAR-BL testing of resistance to third-generation cephalosporin. Consistent with a previous study (Mackenzie et al., 2002), the CAZ hydrolysis assay in our study yielded a high false negative rate in both cultured isolates and BCs, indicating that it might not be appropriate for predicting phenotypic drug resistance. Conversely, the CTX hydrolysis assay successfully identified 100% of ESBL- and 83.3% of AmpC-producing bacterial isolates, suggesting that CTX is a better surrogate marker for the detection of ESBL- and plasmid-mediated AmpC β-lactamase activity. However, the direct detection of ESBL from BCs yielded a CTX hydrolysis sensitivity of only 71.4%, lower than that reported by Jung et al. (2014). We attribute this disagreement to the different logRQ cut-off values used in the two studies (≥0 in Jung et al. vs. ≥0.4 in this study). In fact, 5/7 ESBL producers with false negative results in this study had logRQ values of 0.2–0.4. We note that antibiotic therapy prior to BC collection might also contribute to the occurrence of false negativity in a drug hydrolysis assay. At least two patients from whom the ESBL producers were not detected from BCs by MBT STAR-BL were receiving carbapenem treatment, which might have reduced the viable number of bacterial cells in the BC broths and led to suboptimal CTX and AMP hydrolysis.

Many previous studies involving the mass spectrometric detection of β-lactamase excluded NFGRs, as permeability- and efflux-based resistance mechanisms play major roles in drug resistance in these species (Kumar and Schweizer, 2005). However, NFGRs are important pathogens, and data regarding the ability of MBT STAR-BL to detect drug-resistant NFGR would be useful for routine diagnostic purposes. In our study, the PIP hydrolysis assay correlated perfectly with the phenotypic resistance test for all cultured isolates and BCs. Regarding the MEM hydrolysis assay, MBT STAR-BL correctly identified all carbapenem-resistant NFGRs in cultured isolates. However, in direct BCs, the results for two *A. baumannii* strains harboring *bla*_*OXA*−23, −51_ and one *P. otitidis* strain harboring *bla*_*POM*−1_ were interpreted as indeterminate (logRQ: 0.2–0.4). We attributed the low logRQ values to the low recovery of bacterial cells after extraction and the reported low cell permeability of NFGR (Van Looveren et al., 2004). A previous report suggested that the inclusion of 0.005% SDS in the incubation buffer might have enhanced drug hydrolysis by perforating the membranes of NFGRs (Oviano et al., 2017).

This study also features the unique inclusion of polymicrobial BCs in a full clinical evaluation. The MBT STAR-BL module correctly predicted β-lactam resistance in 14/19 BCs, including those containing ESBL-producing Enterobacteriaceae and carbapenemase-producing NFGRs. These results indicate that the module could help to select the most appropriate antibiotic therapy for patients with polymicrobial bacteremia.

Using the current MBT STAR-BL setting, logRQ values of 0.2–0.4 indicate ambiguous drug hydrolysis and should be reported as indeterminate. However, our findings demonstrate that three AmpC-hyperproducing *E. coli* isolates, six Enterobacteriaceae harboring *bla*_*CTX*−*M*_ and three carbapenemase-producing NFGRs in BCs had logRQ values within this range. A reduction of the cut-off value to 0.2 could increase the detection sensitivities and identify these clinically important organisms, although the specificities would be slightly compromised. This cut-off value reduction led to increases in the sensitivities of the AMP (*E. coli* only), CTX and MEM hydrolysis assays from 92.1, 68.8, and 40% to 95.2, 87.5, and 100%, respectively, for BCs (Table 5). Regarding patient safety, the test aims to rapidly provide information that will lead to effective antibiotic therapy. Accordingly, the sensitivity of the test is more important than the specificity. We therefore recommended eliminating the indeterminate range and setting the logRQ cut-off value at 0.2.

We additionally modified the manufacturer's protocol by using 5% saponin instead of the MALDI Sepsityper kit for BC extraction in MBT STAR-BL test. This is the first study to apply this protocol to the direct detection of β-lactamase-mediated resistance in BCs. Our method yielded similar sensitivities and specificities for AMP, CTX and MEM hydrolysis assay as those reported in previous studies using the Sepsityper kit, if the same logRQ cut-off value was applied (Jung et al., 2014; Oviano et al., 2017). Additionally, the use of 5% saponin (<US$1) is a less expensive option for extraction, compared with the Sepsityper kit (US$17).

In this study, the MBT STAR-BL module was not installed in a clinical laboratory, but rather in an adjacent research laboratory. The time required for sample collection and transportation led to longer turnaround times for MBT STAR-BL measurements in this study (5.2 h) than the value claimed by the manufacturer (2.5 h). Nevertheless, the assay still greatly reduced the turnaround times required to identify β-lactamase-producing organisms in patient-derived BCs. Particularly, drug hydrolysis assays could confirm the presence of ESBL-producing Enterobacteriaceae and carbapenemase-producing NFGRs in BCs at an average of 14 and 48 h before the interim and final reports of routine microbiological processing, respectively, were made available. It should be noted that the time saved by MBT STAR-BL might not be as pronounced as we described for laboratories where the final antibiogram is determined by automated microbroth system or disk diffusion test prepared after short-term incubation (e.g., 6 h) on solid media.

Although MBT STAR-BL cannot provide a full antibiogram, elevated logRQ values suggest the likelihood of ESBL and carbapenemase production. On the other hand, owing to high accuracy in prediction of AMP resistance in *E. coli*, de-escalation of antibiotic therapy might be considered when AMP hydrolysis is negative as determined by MBT STAR-BL. The short assay turnaround time allows the fine-tuning of antibiotic therapy on the same day that a positive blood culture is identified.

There were two weaknesses of this study. First, we could not determine the performance of MBT STAR-BL for the direct detection of carbapenemase-producing Enterobacteriaceae (CPE) in patient-derived BCs, as no CPE were isolated from positive BCs during the study period. Considering that all the archived KPC-, NDM-, and IMP-producing Enterobacteriaceae isolates were correctly detected by MBT STAR-BL in the first part of our study, the analysis module should be capable of identifying CPE regardless of the enzyme type. Second, as it is the first evaluation study of MBT STAR-BL in our locality, the test results were not used to modify the treatment regimen in our hospitals. Therefore we could not determine the impact of the MBT STAR-BL on the patient outcome, such as change in hospital stay, sepsis related mortality, and the cost of care. A large-scale randomized controlled trial study is recommended to further investigate how the implementation of MBT STAR-BL into routine workflow can benefit the patient management.

In conclusion, the MALDI Biotyper system, when equipped with the MBT STAR-BL module, enables the rapid and simultaneous identification of bacterial species and β-lactamase-mediated resistance from BCs and cultured isolates. A reduction of the logRQ cut-off value to 0.2 significantly increased the detection sensitivities for clinically important pathogens. Finally, the low reagent costs and short turnaround time suggest that this test could be used as a tool for early therapeutic guidance in patients with infection.

## Ethics statement

The biological safety approval was obtained from Health, Safety and Environment Office of The Hong Kong Polytechnic University (Ref. number: RSA15096).

## Author contributions

AL, JL, and GS conceived and designed the experiments, performed the experiments, analyzed the data and wrote the paper. RiL, WN, EL, VL, PS, and RR performed the experiments and analyzed the data. KF, WT, RoL, and DT read and approved the final version of the manuscript.

### Conflict of interest statement

The authors declare that the research was conducted in the absence of any commercial or financial relationships that could be construed as a potential conflict of interest.
