# Supplementary material for: Comprehensive Evaluation of the MBT STAR-BL Module for Simultaneous Bacterial Identification and β-Lactamase-Mediated Resistance Detection in Gram-Negative Rods from Cultured Isolates and Positive Blood Cultures
Source: Front Microbiol. 2018 Feb 23;9:334. doi: 10.3389/fmicb.2018.00334 (PMC5829630; doi:10.3389/fmicb.2018.00334)
Supplement: Supplementary file 1 [file Table1.PDF]

Table S1. Identification results of monomicrobial BCs and the respective subcultured isolates by MALDI Biotyper (*n = 134*)

MALDI-TOF identification (no. of strains)	No. of strains identified from blood cultures			No. of strains identified from isolates		
	**Species level [a]**	**Genus level only [b]**	**No reliable ID [c]**	**Species level [a]**	**Genus level only [b]**	**No reliable ID [c]**
**Enterobacteriaceae (126)**						
Escherichia coli (77)	59	14	4	77	0	0
Klebsiella pneumoniae (32)	18	7	7	31	1	0
Klebsiella oxytoca (1)	0	1	0	1	0	0
Proteus mirabilis (5)	4	1	0	5	0	0
Salmonella sp. (1)	1	0	0	1	0	0
Morganella morganii (2)	2	0	0	2	0	0
Enterobacter cloacae (2)	0	2	0	2	0	0
Citrobacter freundii (3)	1	1	1	3	0	0
Pluralibacter gergoviae (1)	0	0	1	1	0	0
Raoultella ornithinolytica (2)	0	0	2	2	0	0
**Total Enterobacteriaceae**	85	26	15	125	1	0
**Non- fermentative GNR (8)**						
Acinetobacter baumannil (4)	2	2	0	4	0	0
Pseudomonas aeruginosa (1)	0	0	1	1	0	0
Pseudomonas otitidis (1)	0	1	0	1	0	0
Stenotrophomonas maltophilia (2)	0	0	2	2	0	0
**Total Non- fermentative GNR**	2	3	3	8	0	0
**Total organisms**	87	29	18	133	1	0

[a] Identifications at species confidence level (Bruker log(score) ≥2.0).
[b] Identifications at genus confidence level only (Bruker log(score) 1.70-1.99).
[c] Not reliable identification but MALDI-TOF identification was matched with conventional bacterial identification (Bruker log(score) 0.00-1.69).
